# Impact of wilting and additives on fermentation quality and carbohydrate composition of mulberry silage

**DOI:** 10.5713/ajas.18.0925

**Published:** 2019-05-28

**Authors:** Ying Chao Zhang, Xue Kai Wang, Dong Xia Li, Yan Li Lin, Fu Yu Yang, Kui Kui Ni

**Affiliations:** 1 Department of Grassland Science, College of Animal Science and Technology, China Agricultural University, Beijing 100193, China; 2 Beijing Sure Academy of Biosciences Co., Ltd., Beijing 100085, China; 3 College of Animal Science and Technology, Jilin Agricultural University, Changchun 130118, China

**Keywords:** Wilting, Additives, Mulberry, Non-structural Carbohydrate, Structural Carbohydrate

## Abstract

**Objective:**

The objective of this study was to investigate the effects of wilting and additives on the fermentation quality, structural and non-structural carbohydrate composition of mulberry silages.

**Methods:**

The selected lactic acid bacteria strains *Lactobacillus plantarum* ‘LC279063’ (L1), commercial inoculant Gaofuji (GF), and *Trichoderma viride* cellulase (CE) were used as additives for silage preparation. Silage treatments were designed as control (CK), L1, GF, or CE under three wilting rates, that is wilting for 0, 2, or 4 hours (h). After ensiling for 30 days, the silages were analyzed for the chemical and fermentation characteristics.

**Results:**

The results showed that wilting had superior effects on increasing the non-structural carbohydrate concentration and degrading the structural carbohydrate. After ensiling for 30 days, L1 generally had a higher fermentation quality than other treatments, indicated by the lower pH value, acetic acid, propionic acid and ammonia nitrogen (NH_3_-N) content, and the higher lactic acid, water soluble carbohydrate, glucose, galactose, sucrose, and cellobiose concentration (p<0.05) at any wilting rate. Wilting could increase the ratio of lactic acid/acetic acid and decrease the content of NH_3_-N.

**Conclusion:**

The results confirmed that wilting degraded the structural carbohydrate and increased the non-structural carbohydrate; and L1 exhibited better properties in improving fermentation quality and maintaining a high non-structural carbohydrates composition compared with the other treatments.

## INTRODUCTION

In the recent years, much research has been devoted to exploiting new forage resources to face the challenge of the rapid development of the livestock industry [1]. Consequently, mulberry (*Morus alba* L.) is receiving increasing attention as a feed resource due to its high yield (40 to 60 tons per ha per year), crude protein (CP) content (18% to 24%) and digestibility (75% to 85%) [2]. Besides, mulberry can grow in temperate, subtropical and tropical regions. However, mulberry grows very fast and deteriorates quickly under the field condition, which means it needs proper storage facilities.

Ensiling is a common way to preserve fresh forages or crops. During ensiling process, lactic acid bacteria (LAB) convert water soluble carbohydrate (WSC) into organic acids, mainly lactic acid, subsequently the acid environment inhibits the growth of undesirable microorganism [3]. However, the natural silage quality of mulberry is usually low because of its relatively low WSC [4]. To improve the fermentation quality, various biological and chemical additives such as LAB and cellulase (CE) have been developed. The addition of LAB especially homofermentative ones could stimulate the lactic acid production and accelerate the rate of pH drop during the early stage of silage fermentation. The CE addition might be beneficial for degradation of the cell walls, which could contribute to releasing fermentable substrate for LAB growth. Except additives, wilting is often recommended to restrict the clostridia growth and the proteolysis [5].

Until now, although a number of studies have been carried out to evaluate these additives and wilting on improving the fermentation quality of forages, little information is available on silage quality of mulberry. Besides, the WSC and fibre fraction composition is important in improving the nutrition digestibility and rumen health of ruminants [6], while there is shortage of knowledge on the effect of additives and wilting on the structural or non-structural carbohydrate concentration variation.

Therefore, this study was conducted to assess the effects of LAB inoculants, CE or wilting on fermentation quality, structural and non-structural carbohydrate concentrations in mulberry silage, and to provide more detailed information about mulberry for ensiling.

## MATERIALS AND METHODS

### Forage harvest and silage preparation

Whole-crop mulberry was harvested in the rainy and hot period 8th July 2017 at the Teaching Experiment Field of China Agricultural University, Zhuozhou, Hebei, China (39.29N, 115.58E, elevation 50 m, annual mean temperature 13.2°C, average annual precipitation 554.14 mm), and the direct-cut mulberry was wilted in the field for 0 hour (h), 2 h, or 4 h. Whole-crop mulberry was harvested at the second cut, approximately 1.2 to 1.5 metre high, 2-year-old, leaving a stubble of 15 cm. The yield of mulberry is 32 t per ha in one year, and the harvest time is juvenile stage. The leaf-stem ratio of material is 2.19 on fresh matter (FM) basis, and the leaf-stem ratio is 1.37 on dry matter (DM) basis. Then, the materials were cut into 1 to 2 cm immediately with a green feed chopping and kneading beater (9QCJ-4, Shijiazhuang Xinnong Machinery Co., Ltd., Hebei, China). Baseline chemical and microbiological characteristics of the pre-ensiled mulberry samples are shown in Table 1.

The materials were prepared with inoculated additives and CE. Among the additives, inoculated additives were diluted with sterile water and mixed homogeneously into the chopped forage; then, they were inoculated at a concentration of 6 log colony forming units (cfu)/g of FM. At the same time, the same volume water (1 mL) was added to the control (CK) silage. One additive was commercial Gaofuji Zhuanglemei (GF, Sichuan Gaofuji Biotechnology Co., Ltd. Sichuan, China), which contained *Lactobacillus plantarum* (*L. plantarum*) and a 10% concentration of CE. Another additive was CE produced by *Trichoderma viride* and obtained from Sinopharm Chemical Reagent Co., Ltd. (Shanghai, China). The third additive was the isolated strain L1 selected from mulberry silage and was identified as *L. plantarum*. This strain can grow in de Man, Rogosa and Sharpe (MRS) broth under the conditions of pH between 3.0 and 9.0, temperature between 5°C and 50°C, and 6.5% sodium chloride; finally, the 24 h pH was 3.60. The nucleotide sequence for the 16S rRNA gene of L1 has been deposited with GenBank, and accession numbers of L1 is LC279063.

Mulberry silages were treated as follows: i) CK, control without additive, ii) L1, *L. plantarum* ‘LC 279063’, applied at 1×10^6^ cfu/g of FM, iii) GF, commercial inoculants, applied at 1×10^6^ cfu/g of FM, iv) CE, applied at a concentration of 150 U/g, these treatments were conducted with no wilting (wilting 0 h), wilting 2 h, and wilting 4 h. Between each two silage treatments, gloves were changed to prevent bacterial contamination, and 150 g materials were packed into plastic bags (20×30 cm). Then, air was removed using a vacuum sealer (DZ-280/2SE, Furuide machinery CO. LTD, Shandong, China). The experiment was measured with 3 replicates in a completely randomized design. The plastic bags were stored at ambient temperature for 30 days.

### Microbial analyses with plate count method

Ten grams of pre-ensiled forages and silages was sampled and blended with 90 mL of sterilized water and serially diluted in sterilized distilled water from 10^−1^ to 10^−5^. The number of LAB was measured by the plate count method using MRS agar incubated at 30°C for 48 h in an anaerobic box (TE-HER Hard Anareobox, ANX-3; Hirasawa Ltd., Tokyo, Japan). The yeast, mold and coliform bacteria was also measured by the plate count method. The yeast and mold was counted on rose bengal medium agar after incubation at 28°C for 48 h, and the coliform bacteria were counted on eosin-methylene blue agar after incubation at 37°C for 48 h. All microbial data were log10 transformed for FM calculations. All media were obtained from Beijing Aoboxing BIO-TECH Co., Ltd., Beijing, China.

### Fermentation and nutritive characteristics

Immediately after the bags were opened, ten grams of each silage sample was homogenized in a blender with 90 mL of distilled water for 1 min and then filtered through four layers of cheesecloth and three layers of filter paper as described by Zhang et al [7]. The filtrate was used to measure ammonia nitrogen (NH_3_-N), the organic acid content and the pH value (FiveEasy 20K; Mettler-Toledo International Inc., Greifensee, Switzerland). NH_3_-N was analyzed with the phenol-sodium hypochlorite method [8]. The organic acid content included lactic acid, acetic acid, propionic acid, and butyric acid and was measured via high performance liquid chromatography (HPLC) (column: Shodex RS Pak KC-811; Showa Denko K.K., Kawasaki, Japan; detector: DAD, 210 nm, SPD-20A; Shimadzu Co., Ltd, Kyoto, Japan; eluent: 3 mmol/L HClO_4_, 10 mL/min; temperature: 50°C).

The residue material was oven-dried at 65°C for 48 h to measure the DM, and then ground to pass through a 1-mm screen and stored at room temperature for analyses of chemical composition. The WSC content was determined with the anthrone method [9]. Nitrogen was determined using the Kjeldahl procedure method (FOSS Kjeltec 2300, Copenhagen, Denmark) [10], and CP was calculated as multiplying total nitrogen (TN) by 6.25. The buffering capacity (BC) of the silage raw materials was measured by suspending 1 g of sample in 100 mL of distilled water for 30 min, followed by titration to pH 4.0 with 0.1 mol/L lactic acid.

### Structural carbohydrate

Neutral detergent fibre (NDF) and acid detergent fibre (ADF) concentrations of pre-ensiled along with silage samples were determined using the method of Van Soest et al [11], and adapted for an ANKOM 2000 Fiber Analyzer (ANKOM Technology, Macedon, NY, USA). In the NDF measurements, a heat-tolerant enzyme and sodium sulphite were also used. Acid detergent lignin (ADL) was determined using 72% H_2_SO_4_ digestion ADF followed by incineration: incineration fraction lost approximated ADL. Hemi-cellulose was calculated as NDF minus ADF, while cellulose was calculated as ADF minus 72% H_2_SO_4_ digestion residue weight.

### Non-structural carbohydrate

A DionexICS3000 system equipped with a pump, an amperometric detector, an automated sampler with a 25-μL injection loop, and a Chromeleon chromatography management system (Dionex, Sunnyvale, CA, USA) was used for sugar identification and quantification. An analytical CarboPac PA10 pellicular anion-exchange resin column (250 by 4 mm) proceeded by a CarboPac PA10 guard column (50×4 mm) was used for sugar separation. A 20-μL aliquot of each sugar sample was diluted with 4,980 μL of distilled water before ion chromatography injection. The monose were eluted with 25 mM NaOH at a flow rate of 1.0 mL/min, and then the oligosaccharides were eluted with 150 mM NaOH. The mobile phase was prepared by diluting carbonate-free HPLC grade 50% (w/w) stock solution in distilled water, filtering with a 0.45-μm membrane, and degassing with compressed nitrogen gas for 30 min before loading.

### Calculations and statistical analysis

Data were analyzed via two-way analysis of variance to evaluate the effects of silage additives (A), wilted time (W) and their interaction (A×W) on chemical composition and fermentation products of mulberry silages. The means were then compared to determined significance using Duncan’s multiple range method. All statistical analyses were performed using the general linear model procedure with SAS 9.0 software (SAS Institute, Cary, NC, USA, 2002). Significance was declared at p<0.05 unless otherwise noted. OriginPro 2017 was used to make the figures.

## RESULTS

### Chemical and microbial characteristics prior to ensiling

The DM content of direct-cut (wilted 0 h) mulberry was 289.4 g/kg, and wilting of 2 h and 4 h increased the DM content to 387.6 g/kg and 462.6 g/kg, respectively (Table 1). Compared with non-wilted materials, the higher WSC content or BC value were found in wilted materials. However, the wilting decreased the CP content, and increased the galactose and cellobiose content significantly (p<0.05). The principal sugars were glucose and sucrose in mulberry. The counts of coliform bacteria were higher (p<0.05) in wilted materials, and the LAB and molds counts were slightly lower (not significant).

### Fermentation quality and microbial population after ensiling

After 30 days ensiling, the pH value, the contents of propionic acid and NH_3_-N, and the ratio of lactic acid/acetic acid was significantly affected by A, W, and A×W (p<0.05) (Table 2). Marked increase in pH was observed in CK- and CE-treated silages with increasing wilting times, while the L1-treated silages keep the pH stable at three wilting rates, and the pH in L1-treated silages were lower (p<0.05) than the other treatments. The lactic acid and acetic acid concentrations of mulberry silages were significantly affected by A or W, but no A×W interaction was observed (p = 0.0936, p = 0.0691, respectively). Significant decrease in lactic acid was recorded in CK-, GF-, and CE-treated silages with increasing wilting times; additionally, wilted silages exhibited lower acetic acid concentration in all treatments than non-wilted silages (p<0.05). The higher ratio of lactic acid/acetic acid was found in L1-treated silage at three wilting rates, and that in 4 h-wilted silages were higher than non-wilted silages when silages were treated with L1 or GF. Wilting decreased the NH_3_-N content significantly (p<0.05), and the lowest NH_3_-N content was observed in the L1-treated silages at any wilted conditions.

The LAB counts were significantly affected by the factor A and W (p<0.05), but not their interaction (p = 0.0867) (Table 3). The LAB counts in 2 h- and 4 h-wilted silages were higher (p<0.05) than that in the 0h-wilted silages, when treated with CK or L1. No coliform bacteria, yeast or molds were detected after ensiling for 30 days at any treatments.

### Crude protein concentration after ensiling

The CP concentration was significantly affected by the factor W (p<0.05), but not A or their interaction (Table 4). In the CK- and GF- treated silages, the CP concentration had no significant difference among three wilted times. The CP concentration in non-wilted silages was higher than 2 h- or 4 h-wilted silages when treated with L1 or CE. In non-wilted silages, the CP concentration in L1-treated silages was higher than that in CK- or GF-treated silages.

### Dry matter and structural carbohydrates

**Figure 1:**
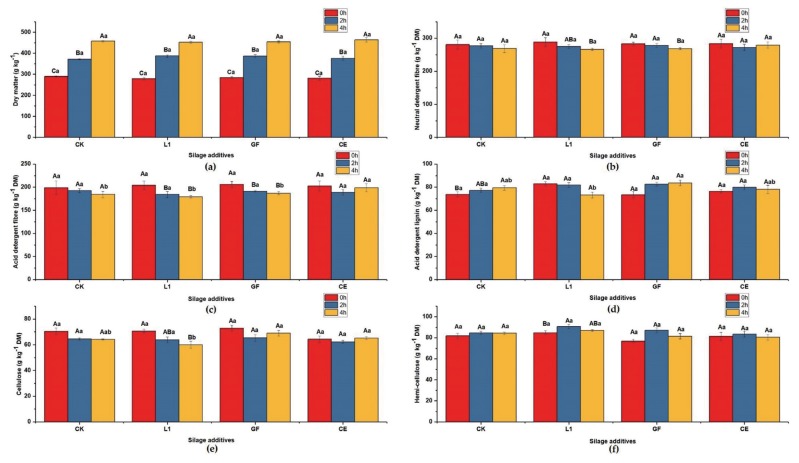
Effect of wilting and additives on (a) dry matter, (b) neutral detergent fibre, (c) acid detergent fibre, (d) acid detergent lignin, (e) cellulose, (f) hemi-cellulose of mulberry silages. CK, silages without additive; L1, isolated LAB strain *Lactobacillus plantarum* ‘LC365281’; GF, commercial inoculant; CE, cellulase; 0 h, 0 h-wilted silages; 2 h, 2 h-wilted silages; 4 h, 4 h-wilted silages; DM, dry matter. Different uppercase letters ^(A–C)^ means differ significantly from each wilted-treated silage of the same additives, and different lowercase letters ^(a–b)^ means differ significantly from each additives in the same wilted-treated silage (p<0.05).

The DM, NDF, ADF and cellulose concentrations were significantly affected by the factor W (p<0.05), but not A or their interaction (Table 5). The DM contents in non-wilted, 2 h-wilted, and 4 h-wilted silages were 271.63 to 293.49 g/kg, 366.25 to 394.62 g/kg, and 445.83 to 473.27 g/kg, respectively (Figure 1a). Additionally, for L1- or GF-treated silages, the NDF and ADF contents in 4h-wilted silages was lower (p< 0.05) than in 0 h- or 2 h-wilted silages (Figure 1b, c). However, CE had no effect on the concentrations of NDF, ADF, and cellulose (Figure 1b, c, d). The factor A, W, or A×W had no effect on ADL or hemi-cellulose (Table 5, Figure 1e, f).

### Non-structural carbohydrates

**Figure 2:**
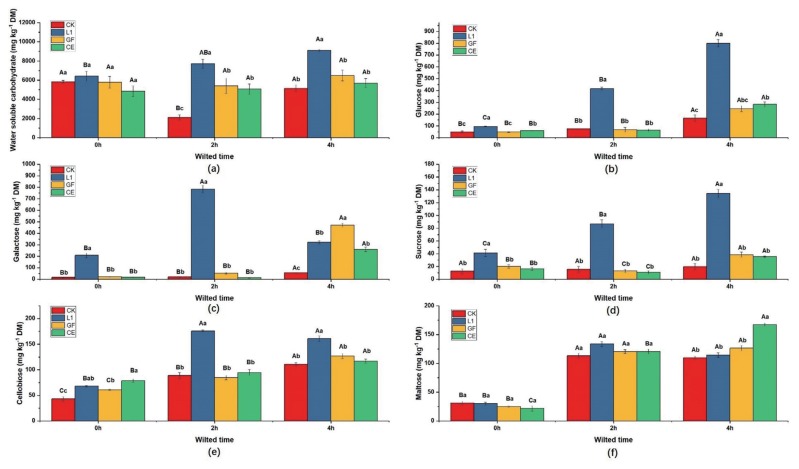
Effect of wilting and additives on (a) water soluble carbohydrate, (b) glucose, (c) galactose, (d) sucrose, (e) cellobiose, (f) maltose of mulberry silages. CK, silages without additive; L1, isolated LAB strain *Lactobacillus plantarum* ‘LC365281’; GF, commercial inoculant; CE, cellulase; DM, dry matter. Different uppercase letters ^(A–C)^ means differ significantly from each wilted-treated silage of the same additives, and different lowercase letters ^(a–b)^ means differ significantly from each additives in the same wilted-treated silage (p<0.05).

After ensiling for 30 days, the WSC, glucose, galactose, sucrose, cellobiose and maltose were significantly affected by the factor A, W, and A×W (Table 5). In the silages without additives (CK), the WSC contents was lower in 2 h-wilted than non-wilted or 4 h-wilted silages (Figure 2a). In 2 h and 4 h wilted material, the higher WSC concentrations were found in L1-treated silages. The glucose contents were significantly increased by wilting (Figure 2b). The glucose contents in L1-treated silages were higher than other treatments (p< 0.05) at three wilting rates. Wilting increased the galactose content when treated with CK, GF, or CE (p<0.05) (Figure 2c). The galactose concentration in silages treated with L1 was higher than other treatments in the silages wilted for 0 h or 2 h. On the other hand, higher galactose content was detected in GF-treated silages when the materials were wilted for 4 h. The L1-treated silages had higher (p<0.05) sucrose concentration than the other treatments under three wilting rates (Figure 2d). When silages were treated with GF or CE, the sucrose concentration ranked as wilted 2 h<wilted 0 h< wilted 4 h; when silages were treated with L1, the sucrose concentration ranked as wilted 0 h<wilted 2 h<wilted 4 h; additionally, silage without additives (CK) had similar sucrose concentration under the wilted conditions. Wilting increased the cellobiose concentration in all treatments, and the cellobiose contents in L1-treated silages were higher than the other treatments at 2 h or 4 h wilted conditions (Figure 2e). The maltose content was significantly lower in the 0 h-wilted silages than the 2 h- or 4 h-wilted silages (Figure 2f). When the mulberry was wilted for 4 h, the maltose content in CE-treated silages was higher than the other treatments.

## DISCUSSION

Ensiling is initiated by LAB in the process of fermentation where LAB is using WSC as its energy and carbon source. Therefore, WSC is essential in producing adequate acidification to achieve well-preserved silages, and its recommended level of concentration is 60 to 70 g/kg of DM [7]. The WSC concentrations in 0 h-wilted, 2 h-wilted, and 4 h-wilted mulberry were 69,234, 77,197, and 78,486 mg/kg of DM in this study, respectively. That meets the recommended minimum level of WSC concentration. The previous researches revealed that glucose, sucrose and fructose could be used by most species of LAB [12], and the main WSC tested in this study are sucrose (52,811.4 mg/kg of DM) and glucose (9,533 mg/kg of DM), which indicates that the LAB in this study had sufficient substrate for fermentation. Additionally, WSC is also a limiting factor in the utilization of silage nitrogen by ruminants, because the efficiency of capture of N is determined by micro-organisms in the rumen, and the micro-organisms must utilize WSC as available energy [13]. Therefore, the WSC preservation is necessary during ensiling.

In the current experiment, the WSC concentration was significantly affected by wilting. The WSC content was higher in the wilted material (p<0.05), which may be caused by the breaking of the lignin protection in the processing of the raw material (cutting into pieces), and the exposed cellulose or hemicelluloses are hydrolyzed to form fermentable sugars [14]. This could be confirmed by the decrease of cellulose content in the wilted material. Additionally, wilting had significant effects on the CP and BC in this study. The CP content in the wilted material was lower than that in the non-wilted material, and this result is similar with the finding of Zheng et al [5]. The reason might be that the plant cells still survived after harvesting, and the degrading enzymes in cells degraded the proteins into small molecules of ammonia during wilting [15]. Moreover, wilting significantly increased the BC, and this was similar with the result reported by Playne et al [16] that BC could be affected by anion fraction, protein and wilting. Previous research indicates a relatively high BC value may be difficult to ensile [17]. Therefore, compared with non-wilted materials, the wilted materials in this study would be more difficult to ensile.

Besides WSC and BC, there are other factors that can help predict the silage quality before ensiling, and the most important factors are epiphytic LAB counts and DM contents [7]. The observed level of LAB (5.84 log cfu/g of FM) in the current study is higher than the recommended level of 5.0 log cfu/g of FM. However, Zhang et al [17] reported that the dominant LAB in mulberry prior to ensiling was *Enterococcus* and *Lactococcus*, which cannot ensure sufficient fermentation. Therefore, inoculants are necessary for improving the fermentation quality of mulberry, and the LAB strain L1 used in this study, isolated from mulberry silages and identified as *L. plantarum*, had a strong acid tolerance and acid production ability [18].

After 30 days of ensiling, the 0 h-wilted silages (except for the L1-treated silages) had higher lactic acid and acetic acid concentration compared with the 4 h-wilted silages. This result can be explained by the low bacterial activity and fermentation acid concentrations due to lower moisture content [5]. Therefore, wilting can influence the growth and reproduction of LAB which is essential to silage fermentation. It is in agreement with Vendramini et al [19], who reported that higher DM content resulted in lower lactate and acetate contents and higher pH value in bermudagrass silage. In addition, the residual WSC concentration in the 4 h-wilted mulberry was significantly higher than that other two wilting time. The changes in the WSC concentration in the mulberry prior to and after ensiling indicated that more WSC was consumed in the 4 h-wilted silages than that in the non-wilted silages. This may be because low pH value can lead to the acid hydrolysis of fibre [20] and inhibit the growth of undesirable bacteria [3]. This result was in agreement with the study of Zheng et al [5], who concluded that a low DM content always coupled with low WSC, and this is readily subjected to clostridial fermentation.

In addition to wilting, additives are always related to non- structural carbohydrate content. Compared with the other treatments, the addition of L1 can significantly increase the concentrations of WSC, glucose and sucrose at the three wilting rates (p<0.05). In this study, no coliform bacteria, yeast or molds were detected after ensiling, and the counts of LAB in the L1-treated silages were lower than those in the CK-treated silages. Thus, the observed increase of non-structural carbohydrates in the L1-treated silages may be due to both acid hydrolysis [20] and inhibition of undesirable bacterial activity [3].

The silages treated with L1 performed a higher fermenta tion quality than the other treatments at the three wilting rates, which is represented by lower pH value, acetic acid content, propionic acid content and higher lactic acid content. This result may be caused by the good properties of L1 which led to a significant decrease in pH even under low moisture conditions. The number of LAB in L1-treated silage was lower than CK-treated silage, which is probably due to the lower pH in L1-treated silage inhibiting the growth of LAB. In the same time, the inoculated L1 is a homo-fermentative strain, which could efficiently convert WSC into lactic acid. Therefore, the number of LAB in L1-treated silage was lower than CK-treated silage, and the fermentation quality in L1-treated silage was better. The L1-treated silages at the 0 h and 2 h wilting rates contained more galactose and the accumulation of galactose may be partially due to lactose fermentation [21], and this could be proved by the high content of lactic acid in the L1-treated silages. The silages treated with L1 also had a lower NH_3_-N content (21.94 to 45.75 g/kg TN) than the other treatments at the three wilting rates. This result could be attributed to a sharp decrease in the pH value which inhibited aerobic bacteria and clostridia and decreased protein degradation [5]. The NH_3_-N content is a reliable indicator of proteolysis in silages, and for well-preserved silages, the NH_3_-N content is less than 100 g/kg of TN [7]. In our study, the NH_3_-N contents in all the treatments were less than 100 g/kg of TN.

In this study, compared with the material before ensiling, the cellobiose concentration increased after ensiling for 30 days. In addition, the CE-treated silages had a higher cellobiose concentration than the CK-treated silages under the 0 h-wilted condition and had a similar cellobiose concentration with the L1-treated silages. The higher cellobiose content in the silages treated with L1 or CE may be explained by Braun [22] that the cellobiose could be obtained by acidic hydrolysis or enzymatic of cellulose during ensiling.

For the effects of CE in silages, predecessors have reported inconsistent results based on the different characteristics of the silage materials. The CE was found to decrease the pH value and increase the lactic acid content in kudzu [23] and sugarcane top [24]. However, it was found that CE did not improve the fermentation quality or affect the fibre degradability of total mixed ration [25] and oat silage [26]. The inconsistent efficacy of enzymes may be related to crop maturity, moisture, storage time and ensiling conditions (e.g., temperature and pH) [27]. The *Trichoderma viride* CE used in our study has been acknowledged to be effective on various lignocellulosic substrates, and can hydrolyze β1,4-glucan linkages in cellulose [28]. However, *Trichoderma viride* CE had no effects on improving the silage quality and fibre degradation in the current study.

Although the additives had no effects on fibre degradation, the concentrations of NDF, ADF, and cellulose were lower in the 4 h-wilted silages compared with those in the 0 h-wilted silages. That may be due to the degradation occurring in the wilting process. The Nutrients Requirement of Dairy Cattle (NRC) in 1989 required that forage should contain 19% to 21% ADF and 25% to 30% NDF in the cows’ diets to maintain the milk fat. In the current study, the ADF or NDF concentration in all treatments met the requirement of NRC.

Therefore, many parameters assessed in our study indi cated mulberry can be a high quality forage, and wilting had the advantages of improving the non-structural carbohydrate concentration (WSC, glucose, galactose, sucrose, cellobiose) and degrading the structural carbohydrates (neutral detergent fiber) in mulberry. The *L. plantarum* L1 improved the fermentation quality, and could be an effective silage additive for ensiling mulberry even under high DM conditions (462.6 g/kg).

## Figures and Tables

**Table 1:** Chemical and microbial compositions in mulberry prior to ensiling

Items	Wilted time			SEM	p-value

	0 h	2 h	4 h		
Dry matter (g/kg)	289.4^c^	387.6^b^	462.6^a^	3.74	<0.0001
Buffering capacity (g LA/kg DM)	57.47^c^	63.23^b^	65.81^a^	0.73	0.0005
Buffering capacity (mEq/kg DM)	637.3^c^	701.2^b^	729.9^a^	8.09	0.0005
Crude protein (g/kg DM)	179.5^a^	168.6^b^	165.5^b^	0.30	0.0124
Neutral detergent fiber (g/kg DM)	300.0	288.3	275.0	6.81	0.1039
Acid detergent fiber (g/kg DM)	210.0	212.2	205.0	12.25	0.9160
Acid detergent lignin (g/kg DM)	62.28	65.06	66.53	3.12	0.6428
Cellulose (g/kg DM)	74.94	70.43	68.50	2.56	0.265
pH	6.97^a^	7.06^a^	6.79^b^	0.04	0.0073
Lactic acid bacteria (log cfu/g FM)	6.11	6.07	5.68	0.29	0.5471
Coliform bacteria (log cfu/g FM)	5.38^b^	6.47^a^	6.45^a^	0.26	0.0416
Yeasts (log cfu/g FM)	5.68	5.50	5.91	0.16	0.2837
Molds (log cfu/g FM)	5.57	5.02	4.90	0.36	0.4271
Water soluble carbohydrates (mg/kg DM)	69,234^b^	77,197^a^	78,486^a^	4,424	0.0018
Glucose (mg/kg DM)	9,533	9,727	9,820	1,301.3	0.9875
Galactose (mg/kg DM)	112.1^c^	201.3^b^	388.3^a^	12.84	<0.0001
Sucrose (mg/kg DM)	52,811	55,921	56,717	2,229.2	0.4706
Cellobiose (mg/kg DM)	-c	23.65^b^	54.76^a^	0.44	<0.0001
Maltose (mg/kg DM)	384.1	372.4	388.6	21.39	0.8606

SEM, standard error of the mean; LA, lactic acid; DM, dry matter; FM, fresh matter.

a–c Mean in the same row with different superscript letters differ significantly from each other (p<0.05).

**Table 2:** Fermentation characteristics in mulberry silages after ensiling for 30 days

Items	Silage additives (A)	Wilted time (W)			SEM	p-value^1)^		
	
		0 h	2 h	4 h		A	W	A×W
pH	CK	4.26^Ca^	4.40^Ba^	4.56^Aa^	0.02	<0.0001	<0.0001	0.0010
	L1	4.16^Ab^	4.14^Ac^	4.17^Ac^	0.00			
	GF	4.24^Aa^	4.30^Ab^	4.36^Ab^	0.02			
	CE	4.23^Ba^	4.40^Aa^	4.47^Aab^	0.01			
Lactic acid (g/kg DM)	CK	63.89^Ac^	58.00^Ab^	49.11^Bb^	1.01	<0.001	<0.0001	0.0936
	L1	75.63^Aab^	73.86^Aa^	65.80^Aa^	1.90			
	GF	83.06^Aa^	64.60^Bab^	63.97^Ba^	2.07			
	CE	69.20^Abc^	59.85^Bb^	50.11^Cb^	0.62			
Acetic acid (g/kg DM)	CK	18.36^Aa^	16.59^Ba^	13.63^Ca^	0.25	<0.0001	<0.0001	0.0691
	L1	14.71^Ab^	10.35^Bb^	8.10^Bc^	0.41			
	GF	21.27^Aa^	15.32^Ba^	12.62^Cb^	0.39			
	CE	20.53^Aa^	16.74^Ba^	14.29^Ba^	0.41			
Lactic acid/acetic acid	CK	3.49^Aa^	3.50^Ac^	3.61^Ac^	0.07	<0.0001	<0.0001	<0.0001
	L1	5.18^Ca^	7.13^Ba^	8.11^Aa^	0.24			
	GF	3.92^Bb^	4.22^Bb^	5.07^Ab^	0.20			
	CE	3.39^Ab^	3.57^Ac^	3.51^Ac^	0.11			
Propionic acid (g/kg DM)	CK	4.30^Bab^	5.27^Aa^	5.79^Aa^	0.16	0.0009	0.0001	0.0341
	L1	2.43^Bb^	4.05^ABab^	5.44^Aab^	0.34			
	GF	4.24^ABab^	3.20^Bb^	4.91^Ab^	0.25			
	CE	5.11^Aa^	4.87^Aa^	5.80^Aa^	0.18			
NH_3_-N (g/kg TN)	CK	62.13^Aa^	71.08^Aa^	47.60^Ba^	1.97	<0.0001	<0.0001	0.0033
	L1	45.75^Ab^	37.97^Bc^	21.94^Cb^	1.16			
	GF	65.24^Aa^	48.54^Bb^	42.35^Ca^	0.94			
	CE	59.47^Aa^	56.56^Ab^	43.84^Ba^	1.77			

SEM, standard error of the mean; CK, silages without additive; L1, *Lactobacillus plantarum* ‘LC365281’; GF, a commercial inoculant containing *Lactobacillus plantarum*; CE, cellulase; DM, dry matter; NH_3_-N, ammonia nitrogen; TN, total nitrogen.

Mean in the same column ^(a–c)^ or row ^(A–C)^ with different superscript letters differ significantly from each other (p<0.05).

**Table 3:** Count of lactic acid bacteria in mulberry silages after ensiling for 30 days

Items	Silage additives (A)	Wilted time (W)			SEM	p-value		
	
		0 h	2 h	4 h		A	W	A×W
Lactic acid bacteria (log cfu/g FM)	CK	4.66^Bab^	6.08^Aa^	6.46^Aa^	0.33	0.0002	0.0004	0.0867
	L1	3.80^Bb^	4.60^Ab^	5.21^Ab^	0.21			
	GF	4.86^Aab^	5.45^Aab^	5.09^Ab^	0.38			
	CE	5.49^Aa^	5.45^Aab^	5.87^Aab^	0.24			

SEM, standard error of the mean; FM, fresh matter; CK, silages without additive; L1, *Lactobacillus plantarum* ‘LC365281’; GF, a commercial inoculant containing *Lactobacillus plantarum*; CE, cellulose.

Coliform bacteria, yeast and molds were not detected in all treatments, and we did not list them in the table.

Mean in the same column ^(a–c)^ or row ^(A–B)^ with different superscript letters differ significantly from each other (p<0.05).

**Table 4:** Crude protein concentration in mulberry silages after ensiling for 30 days

Items	Silage additives (A)	Wilted time (W)			SEM	p-value		
	
		0 h	2 h	4 h		A	W	A×W
Crude protein (g/kg DM)	CK	170.7^Ac^	167.5^Aa^	164.4^Aa^	1.03	0.2880	0.0009	0.1841
	L1	180.1^Aa^	166.8^Ba^	166.3^Ba^	1.72			
	GF	173.8^Abc^	168.4^Aa^	155.7^Aa^	6.86			
	CE	176.7^Aab^	169.4^Ba^	166.3^Ba^	1.54			

SEM, standard error of the mean; CK, silages without additive; L1, *Lactobacillus plantarum* ‘LC365281’; GF, a commercial inoculant containing *Lactobacillus plantarum*; CE, cellulose.

Mean in the same column ^(a–c)^ or row ^(A–B)^ with different superscript letters differ significantly from each other (p<0.05).

**Table 5:** Effects of additives and wilting on the DM, structural and non-structural carbohydrates after 30 days ensiling in mulberry silages

Items						Additive mean				Wilted time mean		
	
						CK	L1	GF	CE	0 h	2 h	4 h
Structural carbohydrate	Dry matter (g/kg)					373.0^a^	373.1^a^	375.1^a^	373.7^a^	283.8^C^	380.5^B^	456.9^A^
	Neutral detergent fibre (g/kg DM)					275.5^a^	276.6^a^	276.5^a^	278.5^a^	284.1^A^	275.7^AB^	270.5^B^
	Acid detergent fibre (g/kg DM)					191.8^a^	189.1^a^	194.7^a^	196.7^a^	202.8^A^	189.2^B^	187.2^B^
	Acid detergent lignin (g/kg DM)					79.83^a^	79.31^a^	79.83^a^	78.15^a^	76.62^A^	80.41^A^	78.57^A^
	Cellulose (g/kg DM)					125.5^a^	123.7^a^	124.5^a^	130.5^a^	133.3^A^	123.1^B^	121.8^B^
	Hemi-cellulose (g/kg DM)					83.62^a^	87.50^a^	81.79^a^	81.82^a^	81.23^B^	86.52^A^	83.30^AB^
Non-structural carbohydrate	Water soluble carbohydrate (mg/kg DM)					4,373.0^c^	7,747.3^a^	5,897.5^b^	5,197.1^bc^	5,724.4^B^	5,078.9^B^	6,607.9^A^
	Glucose (mg/kg DM)					96.78^c^	436.9^a^	121.3^bc^	135.5^b^	63.49^C^	156.0^B^	373.4^A^
	Galactose (mg/kg DM)					31.34^b^	438.2^a^	181.4^b^	96.87^b^	65.38^B^	217.9^A^	277.6^A^
	Sucrose (mg/kg DM)					16.00^b^	87.46^a^	23.84^b^	20.83^b^	22.55^C^	31.56^B^	56.98^A^
	Cellobiose (mg/kg DM)					80.83^c^	134.8^a^	90.79^b^	96.62^b^	62.55^C^	111.0^B^	128.8^A^
	Maltose (mg/kg DM)					84.76^b^	92.64^ab^	90.71^b^	103.3^a^	26.97^B^	122.0^A^	129.5^A^
	**Significance of main effects and interaction**											
**DM**	**NDF**	**ADF**	**ADL**	**Cellulose**	**HC**	**WSC**	**Glucose**	**Galactose**		**Sucrose**	**Cellobiose**	**Maltose**
Additives (A)	0.9019	0.9645	0.4319	0.6156	0.3778	0.1476	<0.0001	<0.0001	<0.0001	<0.0001	<0.0001	0.0181
Wilted time (W)	<0.0001	0.0478	0.0019	0.2158	0.0080	0.0981	0.0056	<0.0001	0.0084	<0.0001	<0.0001	<0.0001
A×W	0.0096	0.8397	0.5594	0.0771	0.6908	0.8965	0.0117	<0.0001	0.0014	<0.0001	<0.0001	0.0003

CK, silages without additive; L1, *Lactobacillus plantarum* ‘LC365281’; GF, a commercial inoculant containing *Lactobacillus plantarum*; CE, cellulase; DM, dry matter; NDF, neutral detergent fibre; ADF, acid detergent fibre; ADL, acid detergent lignin; HC, hemi-cellulose; WSC, water soluble carbohydrate.

Different label ^(a–c)^ in the same row means differ significantly from each additive in the same wilted-treated mulberry, and different label ^(A–C)^ means differ significantly from each wilted-treated silage in the same additives (p<0.05).
